# A Transcriptomic Analysis of Laryngeal Dysplasia

**DOI:** 10.3390/ijms25179685

**Published:** 2024-09-07

**Authors:** Fausto Maffini, Daniela Lepanto, Francesco Chu, Marta Tagliabue, Davide Vacirca, Rita De Berardinis, Sara Gandini, Silvano Vignati, Alberto Ranghiero, Sergio Taormina, Alessandra Rappa, Maria Cossu Rocca, Daniela Alterio, Susanna Chiocca, Massimo Barberis, Lorenzo Preda, Fabio Pagni, Nicola Fusco, Mohssen Ansarin

**Affiliations:** 1 Department of Surgical Pathology, European Institute of Oncology IRCCS, 20141 Milan, Italy; 2 Division of Otolaryngology Head and Neck Surgery, European Institute of Oncology IRCCS, 20141 Milan, Italy; 3 Department of Biomedical Sciences, University of Sassari, 07100 Sassari, Italy; 4 Molecular and Pharmaco-Epidemiology Unit, Department of Experimental Oncology, European Institute of Oncology IRCCS, 20141 Milan, Italy; 5 Medical Oncology Division of Urogenital and Head and Neck Tumors, European Institute of Oncology IRCCS, 20141 Milan, Italy; 6 Department of Radiotherapy, European Institute of Oncology IRCCS, 20141 Milan, Italy; 7 Department of Experimental Oncology, European Institute of Oncology IRCCS, 20139 Milan, Italy; 8 Diagnostic Imaging Unit, National Center of Oncological Hadron-Therapy (CNAO), 27100 Pavia, Italy; lorenzo.preda@unipv.it; 9 State University School of Medicine, University of Pavia, 27100 Pavia, Italy; 10 Department of Medicine and Surgery, Pathology, IRCCS Fondazione San Gerardo dei Tintori, University of Milano-Bicocca, 20126 Milan, Italy; 11 State University School of Medicine, University of Milan, 20122 Milan, Italy

**Keywords:** laryngeal dysplasia, cancer biology, cancer treatment

## Abstract

This article describes how the transcriptional alterations of the innate immune system divide dysplasias into aggressive forms that, despite the treatment, relapse quickly and more easily, and others where the progression is slow and more treatable. It elaborates on how the immune system can change the extracellular matrix, favoring neoplastic progression, and how infections can enhance disease progression by increasing epithelial damage due to the loss of surface immunoglobulin and amplifying the inflammatory response. We investigated whether these dysregulated genes were linked to disease progression, delay, or recovery. These transcriptional alterations were observed using the RNA-based next-generation sequencing (NGS) panel Oncomine Immune Response Research Assay (OIRRA) to measure the expression of genes associated with lymphocyte regulation, cytokine signaling, lymphocyte markers, and checkpoint pathways. During the analysis, it became apparent that certain alterations divide dysplasia into two categories: progressive or not. In the future, these biological alterations are the first step to provide new treatment modalities with different classes of drugs currently in use in a systemic or local approach, including classical chemotherapy drugs such as cisplatin and fluorouracile, older drugs like fenretinide, and new checkpoint inhibitor drugs such as nivolumab and pembrolizumab, as well as newer options like T cell therapy (CAR-T). Following these observed alterations, it is possible to differentiate which dysplasias progress or not or relapse quickly. This information could, in the future, be the basis for determining a close follow-up, minimizing surgical interventions, planning a correct and personalized treatment protocol for each patient and, after specific clinical trials, tailoring new drug treatments.

## 1. Introduction

Laryngeal dysplasia is a premalignant lesion that significantly impacts patients’ social life and quality of life. Despite advancements in surgical techniques and minimally invasive surgical treatments, the dysplasia in many cases progresses to laryngeal squamous cell carcinoma (LSCC), with a great number of sequels being related to swallowing and speech disorders due to the disease and repeated treatments.

Therefore, identifying biological alterations in the laryngeal mucosa provides the basis for hypothesizing different therapeutic possibilities compared with surgical intervention, aiming to preserve laryngeal function—an ambitious but desirable objective.

LSCC is a stepwise process, progressing from laryngeal premalignant dysplasia (LDy) to invasive carcinoma [1,2]. The diagnosis of LDy is based on cytoarchitectural changes in the squamous epithelium laryngeal wall. However, the histopathologic grading alone is not sufficient to define the risk of malignant progression towards invasive cancer. It also does not allow for standardized management, which can range from simple observation to biopsy under endoscopic control, radical excision with a cold blade, or transoral laser microsurgery with a CO_2_ laser [3,4]. 

Recently, the risk of malignant progression from LDy to LSCC has been found to be related to a series of immunogenetic mutations and extracellular matrix alterations [5].

We recently reported on the different immunogenetic landscape of LDy progressing to invasive cancer over time (progressing dysplasia, PDy) and LDy not evolving towards LSCC (non-progressing dysplasia, NPDy). We found that the difference observed in the two sub-groups, in terms of risk of malignant progression, was significantly related to transcriptional alterations, thus resulting in an up-regulation or over-expression of some genes (over-expression assumed mRNA transcript expression value of >2-fold). Some of these genes were positively related to an augmented risk of LSCC, while others were more expressed in NPDy, thus having a protective role [5].

Furthermore, tumor-infiltrating lymphocytes and altered expressions of specific genes associated with the tumor–host innate immune system have been shown to be independent risk factors for the risk of malignant progression towards LSCC [5,6].

Moreover, 20% of hyperplastic laryngeal lesions with loss of heterozygosis (LOH) involving p16ink4 and p53 might be considered potentially dysplastic. Hyperplasia does not show the same morphological, cytological, and architectural atypia as low-grade dysplasia and could be underestimated [3,7,8].

The decision making in LDy is well defined and unchanged overtime. The therapeutic treatments considered are surgery, radiotherapy (RT), and in some cases, planned a close follow-up [7]. Therefore, if it was possible to identify PDy and NPDy early, we could use different therapeutic and control planning based on the risk of malignant transformation and propose adequate treatment choices [3].

The histopathological report, including grading and the type of lesion (verrucose, mixed or basaloid), is not enough to recognize an NPDy or a PDy [6,9]. It is necessary to identify prognostic factors that can help find the best personalized therapeutic approach, achieving disease control and avoiding or delaying unnecessary surgical or radiotherapy treatments. Understanding the dysplasia transcriptomic alterations may help define the biological behavior of these premalignancies, assuming possible medical therapies improving patients’ prognosis [3,5,10].

In this work, we aim at providing new insights into LDy transcriptomic alterations for a better understanding of the cancerogenesis-leading mechanisms.

## 2. Results

There are significant differences between the NPDy and PDy. Previous reports highlighted the role of the adaptative–innate immune system and TILs in LDy and advanced LSCC. Specifically, the immune system reaction and extracellular matrix factors (ECMs) greatly influenced patients’ prognosis in advanced laryngeal cancer and the risk of malignant progression from LDy to invasive cancer [5,10].

**Figure 1:**
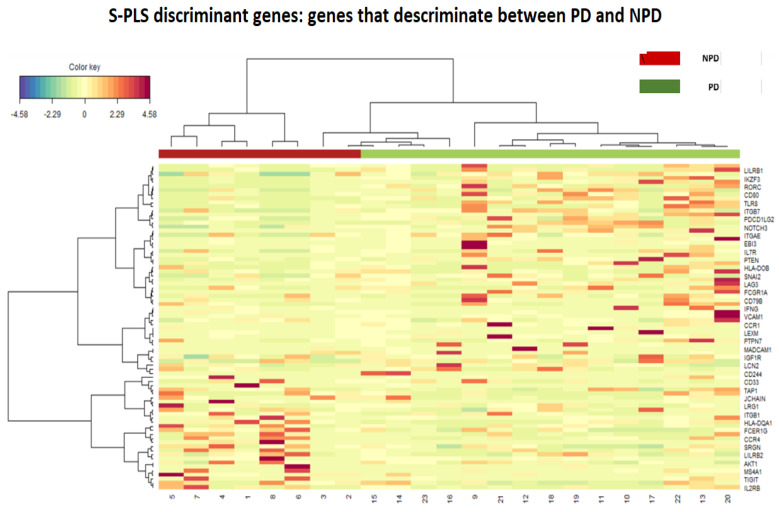
The hierarchical heatmap of the S-PLS discriminant analysis showing the difference in the expression of genes that better discriminate between the PDy (green) and NPDy (red). The color intensity also shows the value of expression. The authors are grateful to Cancers for kindly allowing the use of this table.

**Figure 2:**
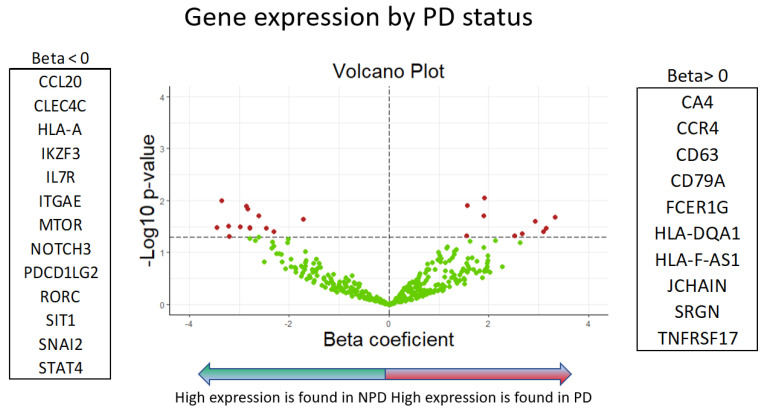
Volcano plot of gene expression by PD (ANOVA models), the red dots indicate gene expression values that show statistical significance, characterised by (*p*-value on the y-axis), versus the magnitude of change (fold change on the x-axis). They are located above the line of significance (horizontal dashed line), allowing us to quickly identify the most biologically significant genes.

**Figure 3:**
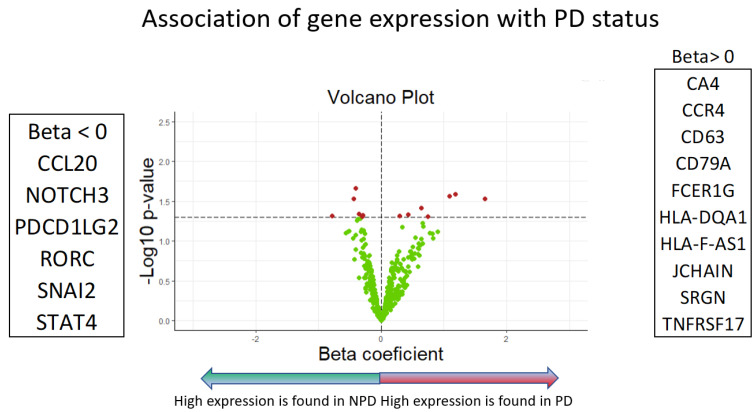
Volcano plot of gene expression association with PDy (Logistic models), also in this image, the red dots indicate gene expression values that show statistical significance, characterised by (*p*-value on the y-axis) versus the magnitude of change (fold change on the x-axis). They are located above the line of significance (horizontal dashed line), allowing us to identify the most biologically significant genes.

Genes belonging to different families with different functions were differentially expressed in NPDy or PDy (Figure 1, Figure 2 and Figure 3).

Many of them were involved in the chemokine signaling, tumor markers, antigen processing, B cell marker, helper T cells, neutrophil, checkpoint inhibitors, and lymphocyte infiltrate. In cases of PDy, there was a more than 2-fold over-expression of genes compared with the NPDy (Table 1).

### 2.1. Dysregulated Genes Involving the Chemokine Signaling

The motif chemokine ligand 20 (*CCL20*) regulating the chemokine signaling was up-regulated in NPDy, while G protein-coupled receptor family 4 (*CCR4*) was over-expressed in PDy.

*CCL20* is a secretory protein involved in inflammation, modulating the immune response and showing chemotactic activity among the lymphocytes and myeloid series. In our cases, this protein was observed to be up-regulated in NPDy. No reports indicated an up-regulation of *CCL20* in the dysplastic lesion, but it has been reported to be up-regulated in lung adenocarcinoma as a poor prognostic marker [11]. In lung adenocarcinoma, *CCL20* promotes tumor progression by increasing the epithelial–mesenchymal transition (EMT), facilitating invasion, and metastasizing. This effect is linked to TGF-β and its receptor *CCR6*. Tao Fan et al. (2022) suggested that the activity of *CCL20* appears to be linked to the tumor’s non-response to immunotherapy when linking to TGF-β, blocking the immune checkpoint receptor, and promoting the metastatic diffusion [11,12]. Qian Song et al. (2020) suggested another pathway in which *CCL20* was up-regulated by *RUNX3*, recruiting the CD8^+^ T cells. [12]. The CC motif chemokine receptor 6 (*CCR6*) was observed to be increased in high TILs in PDy (recruiting the lymphocyte and dendritic cells) and modified the extracellular matrix (ECM). *CCR6* binds with its ligand *CCL20*, promoting cancer progression. The *CCL20–CCR6* axis was involved in neoangiogenesis due to ischemic damage [13,14]. In NPDy, the axis could be inactive, blocking or slowing down the progression of dysplasia. It is noteworthy that *CCL20* was reported to be up-regulated in patients who smoke cigarettes [5,13].

Additionally, the G protein-coupled receptor family 4 (*CCR4*) is a chemokine signaling-related receptor. This receptor for CC motif chemokine is responsible for homeostasis, and it regulates many types of leukocytes, the immune system, and angiogenesis; it was found to be more expressed in PDy. *CCR4* was reported to be over-expressed in many hematological and solid tumors and also in head and neck tumors. Zihang Ling et al. (2022) showed that *CCR4* was over-expressed in tumors with poor prognosis in head and neck squamous cell carcinoma (HNSCC). Their work demonstrates that the axis with its ligand CC motif chemokine ligand 2 (*CCL2*) promotes motility and progression with increased metastatic ability. The authors, by inhibiting this characteristic with siRNA (silencing RNA), reported a lowered ability to exhibit motility, progression, and metastatic potential in HNSCC [15]. The over-expression in PDy could increase the motility of dysplastic cells, thereby enhancing tumor progression.

### 2.2. Major Histocompatibility Complex Dysregulated Genes

The histocompatibility complex *HLA-DQA1* and *HLA-F-AS1* were found to be over-expressed in cases of PDy. The *HLA-DQA1* complex belongs to a major histocompatibility complex (HLA), class II, DQ alpha 1, mRNA. This membrane receptor protein plays a role in presenting antigens to the antigen-presenting cells (APCs) of the immune system for CD4 T cell recognition. There are limited data highlighting the role of *HLA-DQA1* in carcinoma progression. Sheng-Chien Tsai et al. (2011) described the protective role of *HLA-DQA1* in carcinoma development, but they did not find an up-regulation between control and tumor cases [16]. *HLA-DQA1* was reported to be over-expressed in sarcoma, and this over-expression was associated with long-term survival [4]. However, an over-expression of this gene in PDy was observed in the cases under consideration, suggesting that *HLA-DQA1* may attempt to enhance APC functions and present antigens to CD4 T cell but fails, possibly due to a blockage in its pathways.

*HLA-F-AS1* is another major histocompatibility system (HLA-F) antisense RNA 1 transcript variant 1, a long non-coding RNA (lncRNA). This was over-expressed in the cases of PDy under study, suggesting a role in dysplasia progression with a mechanism similar to that observed for *HLA-DQA1* but involving different pathways. This observation aligns with the findings of Huang Y. et al. (2020), where the *HLA-F-AS1* was up-regulated in colorectal adenocarcinoma, promoting carcinoma progression via miR-330-3p. The *HLA-F-AS1* lncRNA FGD5-AS1-miR-330-3p axis was reported to be altered. This axis enhances the resistance of neoplastic cells to 5-fluorouracile drugs (5-FU) via epithelial growth factor receptor (EGFR) in colorectal cancer [17,18].

### 2.3. Dysregulated Genes of B Cell Markers

The B cell marker *TNFRSF17*, *JCHAIN*, and CD79A were observed to be over-expressed and associated with PDy in the cases under consideration.

The tumor necrosis factor receptor superfamily 17 mRNA (*TNFRSF17*), belonging to a superfamily of TNF-receptor, was expressed in mature B lymphocytes. Its significance lies in enhancing the immune response through the nuclear factor kappa-light-chain-enhancer of activated B cell (NF-kB) activation. This transcription factor activates MAPK/JNK and reprograms the B cell [19]. *TNFRSF17* can use a different pathway in the transduction of the signal, employing a tumor necrosis factor receptor-associated factor (TRAF) member family, thereby increasing cell survival and proliferation. 

Another gene that was over-expressed in PDy was a *JCHAIN* (Homo sapiens joining chain of multimeric IgA and IgM, mRNA) [20]. This gene regulates the function of polymeric IgA (pIgA) and pentameric IgM. IgA and IgM are secretory immunoglobulins (SIgs) secreted by mucosal plasma cells associated with lymphoid tissue in the oral cavity (MALT). IgA and IgM were created by plasma cells in the rough endoplasmic reticulum and were synthesized in the Golgi apparatus. When synthesized, the Golgi apparatus formed a dimer of IgA with *JCHAIN*, creating a polymeric IgA (pIgA) and linking the IgM in a pentameric structure. After polymerizing with *JCHAIN*, the pIgA and a pentameric IgM were delivered and secreted into the extracellular matrix. This dimeric pIgA and pentameric IgM strongly binded with their receptor (pIgR) through *JCHAIN* on the epithelial cell. The epithelial cell then transported the pIgA and a pentameric IgM from the basal layer to surface layer for secretion on the luminal surface [21]. Why *JCHAIN* was up-regulated in PDy is unknown. We suppose that the secretory activity of epithelial cells was lost, resulting in the accumulation of pIgA and pentameric IgM, possibly due to increased TILs, as reported in our previous work [5]. We speculate that in PDy, the loss of SIg does not allow for the agglutination and cytolyzing of bacteria, leading to damage on the natural barrier on the surface of the larynx and increasing the possibility of infection. It is known that SIg is important in preventing the infection by agglutinating and cytolyzing the bacteria. The deficiency in the immune system to infection increases the production of IgA and IgM. In dysplastic lesions, blockage and slowing down of the delivery activity of immunoglobulin transport from the basal side to luminal surface favor bacteria proliferation and inflammation [22].

The human immunoglobulin-associated alpha transcript variant 1 gene (CD79A), encoding for a Sig receptor-α on the B cells, is necessary for the antigenic B cell receptor function. According to our data, CD79A is over-expressed in PDy. We speculate that this could be due to the decreased SIg secretion in response to epithelial damage, followed by increased infection. 

Our previous work showed that CD79A was up-regulated and associated with better prognosis in invasive laryngeal carcinoma, and this was related to the stage of the disease; CD79A was more up-regulated in pT1-3 then in pT4. This up-regulation was also demonstrated by Yan Chen et al. (2022) in their work [10,21].

These genes (*JCHAIN* and CD79A*)* were reported to be lower expressed altogether in colorectal carcinoma and in others non-squamous cell carcinoma by Pan J. et al. (2021), suggesting a potential indicator for immunotherapy. The role of the B cell regulatory pattern can improve the immune response against cancer [22,23]. The high expression of *JCHAIN* and CD79A could decrease the immune response in PDy lesions and may be ineffective in preventing dysplasia progression.

### 2.4. Dysregulation of Lymphocyte Infiltrate Genes

In PDy, we observed an over-expression of CD63, *SRGN,* and *FCER1G*, which are markers of lymphocyte infiltration. 

Fc ε-receptor Ig (*FCER1G*) encodes a protein similar to the Fc fragment of γ-Ig and has a pivotal role in infection by activating the phagocytosis in myeloid cells and inducing an antineoplastic activity. The immune system also induces the up-regulation of *FCER1G* in PDy [24,25]. *FCER1G* is a high-affinity IgE receptor and was found to be over-expressed in PDy in our cases. It regulates many aspects of the immune response by binding to many FcR α-chain receptors among CD8^+^T cells, CD4^+^T cells, B cells, macrophages, and dendritic cells (DCs). This boosts the inflammatory response by recognizing neoplastic antigens and providing an innate-like immune response with high cytotoxic activity [26,27]. *FCER1G* was involved in the activation via the neutrophil of collagen, mediated by integrins. Although *FCER1G* dysregulation has been reported in multiple myelomas with a good prognosis [28], in some reports, it’s up-regulation was associated with poor overall survival and disease-free survival in head and neck carcinoma [29,30]. The exact mechanism of *FCER1G* in dysplastic lesions of the head and neck is unknown, but chronic inflammation is suggested as an etiopathogenetic factor, as proposed by Mantovani et al. (2008) [31]. 

CD63 is a surface protein belonging to the transmembrane 4 superfamily, regulating motility, growth, and cell development. Down-regulation of CD63 was associated with metastasis and neoplastic progression in melanoma and other tumors [32,33], as well as increased motility in tumor cells and enhanced matrix-degrading ability. This surface receptor is crucial in ECM control, and its down-regulation boosts ECM degradation linked with interring beta1 [34,35]. Takino T. et al. (2003) demonstrated that CD63 has the ability to link membrane-type 1 matrix metalloproteinase (MT1-MMP) and influence the lysosomal proteolysis [35]. In the early phase of melanoma progression, CD63 was up-regulated; however, during disease progression, there is a down-regulation in CD63, increasing a metastatic potential [34]. In PDy, the up-regulation of CD63 aims to inhibit the development of the metastatic potential and mobility of dysplastic cells while simultaneously inhibiting the proteolysis of ECM against cancer progression.

The serglycin transcript variant 1 mRNA (*SRGN*) is a protein stored in secretory granules of many hematopoietic cells, neutralizing proteolytic enzymes. It was over-expressed in PDy in our cases. In the literature, *SRGN* is an important poor prognostic marker in lung adenocarcinoma negative for TTF1 expression (thyroid transcription factor 1), while in nasopharyngeal carcinoma, it was reported to be highly expressed in metastatic cases [36]. *SRGN* has been associated with monocytes that secrete *SRGN*. The over-expression of *SRGN* in PDy suggests an action against ECM degradation by neutralizing proteolytic enzymes. 

### 2.5. Dysregulated Neutrophil Genes

Carbonic anhydrase 4 (CAIV or CA4) is a mRNA transcriptor codifying for a zinc metalloenzyme. It has catalytic properties that participate in several biological activities, such as collagen and bone resorption, acid–base balance, respiratory function, gastric acid regulation, cerebrospinal fluid regulation, and others. This enzyme was reported to be up-regulated in chronic obstructive pulmonary disease (COPD). Nava et al. (2021), using the immunohistochemical method, found that increased expressions of CA4 in chondrocytes and collagen in patients with COPD were followed by fibrosis and cartilaginous calcification of the bronchial wall. This is because of a higher pH promoted by CA4 and a long-standing lung inflammation [37]. As of our knowledge cutoff date, there is no available literature that directly compares CA4 up-regulation with prognosis and progression in HNSCC and dysplasia. The only work demonstrating a predictive value was found in colorectal carcinoma (CRC), where CA4 and CA1 were predictive factors for prognosis [38]. In our cases of PDy, we speculate that inflammation promotes the production of CA4, leading to collagen degeneration and desmoplasia and, in advanced cases, allowing for the dysplastic progression to infiltrate the carcinoma by altering the ECM. However, further research is needed to understand the specific role of ECM in dysplasia.

### 2.6. Dysregulated Helper T Cell Genes

The signal traducer and activator of transcription 4 mRNA (*STAT4*) was found to be up-regulated in NPDy. This gene is linked to a surface receptor mediated by interleukin-12 (IL-12), which activates its transcription. IL-12 plays a key role in regulating T-helper cells. According to W.E. Thierfelder et al. (1996), IL-12, through INF-γ, contributes to the defense against bacterial and parasitic infection. Disruption of IL-12 and INF-γ deprives mitogenesis and enhances the cytolytic property of natural killer cells (NKs) due to the loss of thyrosinase phosphorylation activation in T lymphocytes [39,40,41,42]. We hypothesize that the up-regulation of *STAT4* activates CD4^+^T helper cells and increases NK cytolytic function, preventing neoplastic growth.

Retinoic acid-related orphan receptor C mRNA (*RORC*) is a DNA-binding protein receptor located in the nuclear membrane belonging to a subfamily of nuclear hormone receptors (NR1). The function of this gene is still unknown, but in vitro studies indicate its activity in lymphoid and thymus development, particularly in the subtype of CD4^+^ T cells known as T-helper 17 (Th17) [41,42].

While only one study reported a better prognosis for *RORC* up-regulation in ovarian surface carcinoma, indicating a role in regulating and increasing pro-apoptotic activity and suppressing the neoplastic growth, a down-regulation of *RORC* is associated with poor prognosis in bladder carcinoma. An over-expression of *RORC* has been linked to the down-regulation of PD-L1 mRNA [43,44,45]. The up-regulation of *RORC* in the cases under study enables the Th17 cell activity, delaying dysplastic progression and potentially down-regulating PD-L1 [45]. Our cases did not exhibit an up- or down-regulation of PD-L1 mRNA. Further studies are necessary to understand the mechanisms of *RORC* action in laryngeal dysplasia.

### 2.7. Dysregulated Genes Linked to Checkpoint Inhibitors

Programmed cell death 1 ligand 2 mRNA (*PDCD1LG2*) was found to be up-regulated in NPDy. Checkpoint inhibitors have emerged as crucial drugs in the treatment of HNSCC. PD-L2 works synergistically with PD-L1 in the control of the disease. PD-L2 was negatively correlated with CD4^+^ and CD8^+^ T cells through DCs, which belong to APCs and its ligand PD-L2 in T cells [46,47]. Research by Yearley J.H. et al. (2017) demonstrated that PD-L2′s prevalence, independently of PD-L1, predicts the clinical response to pembrolizumab in neoplastic, stromal, and immune cells, leading to long progression-free survival (PFS). The association between PD-L1 and PD-L2 has been shown to improve the response to pembrolizumab by up to 27.5% [48]. The only difference between this study and Yearly’s was the method employed to evaluate PD-L2. We evaluated the up-regulation of mRNA, while Yearley evaluated the expression of PD-L2 with an immunohistochemical assay [48].

Moratin J. et al. (2019) showed an over-expression of PD-L2, evaluated through immunohistochemistry, in patients with HNSCC, which is predictive of a poor prognosis [49]. Another mechanism linked to *PDCD1LG2* involves its fusion with CD274 (PD-L1). Bossi P. et al. (2017) reported that the presence of fusion genes CD274-*PDCD1LG2*, found in 50% of their cases, was associated with a shorter progression-free survival [50].

This unexpected result opens the door to interesting speculations, suggesting that immunotherapeutic drugs such as nivolumab and/or pembrolizumab could be used in H&N dysplasia therapy, particularly when the surgery fails to control the disease. This concept holds promise and should be investigated further to explore its potential in clinical applications.

### 2.8. Dysregulated Tumor Genes

The notch receptor 3 mRNA (*NOTCH3*) gene belongs to the family of transmembrane receptors that includes *NOTCH* gene 1, 2, and 4 and three transmembrane receptors, that are important in the embryonic development of mammals. The alteration of NOTCH3 was observed in patients with inherited cerebral autosomal-dominant arteriopathy disease with subcortical infarct and leukoencephalopathy syndrome (CADASIL), characterized by cerebral developments dysfunction [51]. In NPDy cases, this gene was up-regulated. Zhang Y.Q. et al. (2021) reported that *NOTCH3* up-regulation inhibits the AKT–mTOR pathway and regulates the PTEN expression in breast carcinoma [52]. This *NOTCH3*-mediated pathway inhibits the proliferation and malignancy in breast cancer [50], suggesting a role for *NOTCH3* in delaying cancer progression in dysplastic lesions of the larynx. Additionally, the up-regulation of *NOTCH3* was reported in SCC, and it has been associated with resistance to the 5-FU drug and reduced sensitivity to cisplatin in nasopharyngeal carcinoma [52,53,54,55].

Regarding *SNAIL* family transcriptional repressor 2 mRNA (*SNAI1* and *SNAI2*), a member of the C2H2 zinc finger transcription factors, *SNAI2* was found to be up-regulated in our cases of NPDy. In a previous study, it was reported that *SNAI1* was up-regulated in PDy in high TIL cases [5]. This gene is involved in epithelial–mesenchymal transition (EMT), a process in which the differentiating cell acquires stem cell characteristics and the ability to differentiate into various cell lineages [47,56]. This transition results in a hybrid cell with a mesenchymal and epithelial phenotype.

Slug (*SNAI2*) belongs to the same family; however, it has a distinct role in regulating cells with stem-like features by inhibiting them. It’s up-regulation and/or over-expression could inhibit EMT due to *SNAI1* [56]. This may occur because SNAI2 exerts its regulatory influence over the epithelial cell adhesion molecule (EPCAM), a molecule associated with stem cells during embryogenesis [56,57].

## 3. Discussion

This paper aimed to answers and insights on the following: (1) the observed differences in dysplastic lesions related to alterations of transcriptional genes belonging to the innate immune system; (2) how these alterations could be used for medical therapy; (3) the role of inflammation, due to local bacterial infection, in increasing neoplastic progression; (4) The differences between PDy and NPDy related to the host’s innate immune system which could be subjects for future medical therapy when standard treatments, such as surgery or RT, fail to control the disease.

These results suggest that every dysplasia can be considered a unique disease with different biological alterations involving the dysplasia itself, the host immune system, the ECM, and bacterial infection. These observations in the progression of dysplasia were reported by Chai Peng Gan et al. (2022), who noted that dysplastic lesions show different evolutions due to different transcriptional analyses [58].

**Figure 4:**
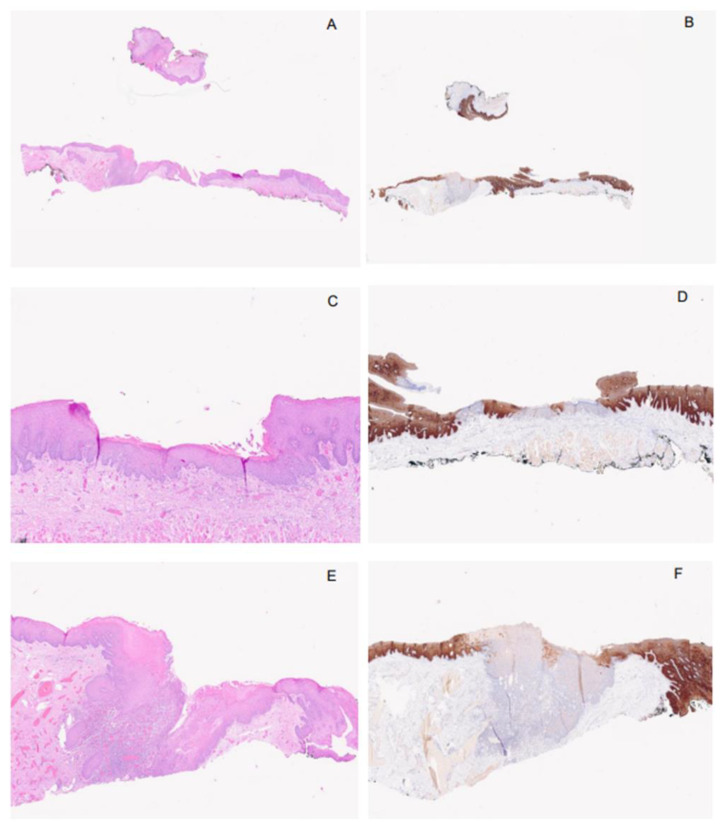
Field cancerization, (**A**–**F**): This example image shows the “field cancerization” in a mucosal epithelium of the oral cavity; the H&E stain in the left of the image shows how the pathologist observes the dysplasia (**A**,**C**,**E**). The overall image showed (in (**A**)) has four points of dysplasia; one of these easier to evaluate morphologically (in (**E**)), while the others are difficult to differentiate from a hyperplastic reactive epithelium that show the same alteration (hyperkeratosis with hypergranulosis and elongation of the rete ridge) of a low-grade dysplasia (in (**C**)). The right images show how staining with keratin 13 (according to the factory guideline Supplementary S3) helps the pathologist to differentiate a hyperplastic-reactive-normal epithelium (brown staining) from dysplasia (unstained epithelium) (in (**B**,**D**,**F**)). Of interest are the foci of dysplasia, where the normal epithelium is spaced; this is a situation of a large number of cases where the dysplastic epithelium involves the entire epithelial surface as a “leopard patch”. This distribution of dysplasia creates a difficult therapeutic choice and leads to difficult recovery and control of the disease. While the image (in (**E**)) was observed by the physicians as a leukoplakia “white spot”, the others (in (**C**)) could disappear at the physician’s evaluation. The same morphological alteration can affect the laryngeal epithelium with the same histological and immunohistochemical alteration reported here in the oral cavity. The magnification of all images is 100X (Objective 5× and Eyepiece ×20).

It is well established that oral cavity and larynx tumors arise after precancerous damage. These types of alterations involving the upper areal digestive system were termed “Field Cancerization” and have been demonstrated since 1953 by Slaughter et al. [59] (Figure 4A–F). The genesis of dysplasia is also well established and attributable to the LOH of 9p21 involving p16^ink4a^ and 17p13 involving p53 [59,60], both of which are proteins involved in cell cycle control and pro-apoptotic factors. From a histopathological point of view, this genetic LOH causes overgrowth, leading to a change in the epithelial morphology. The staining properties of cells become darker due to an increase in nuclear DNA. This alteration has also been observed in hyperplastic reactive lesions [1,2,9].

Defining the degree of dysplasia could be subject to many differences in interpretation by the pathologist and may be prone to errors, especially in the case of low-grade dysplasia and hyperplastic lesions (Figure 4A–F). However, it remains as the only information about tumor progression and the type of tumor that might develop [6]. Actually, the type of dysplasia (I.E basaloid- vs. simplex-type) shows different progression timing and histopathological correlation [6]. This results in different dysplastic lesions, each with different prognoses and treatment results [3,6].

Following the microsatellite instability, Jang S. et al. (2001) showed that the dysplasia may involve different clonal cells [61,62,63], reinforcing the idea of a multiple lesion process in neoplastic premalignant lesions that are “ab-initio” different [63]. In HNSCC, the dysplastic lesions progress to invasive carcinoma in 17.6% of cases at 4 years and 36,4% at 8 years [64]. This has a significant impact on therapeutic choices. In fact, we know that “Field Cancerization” can reduce the recovery chance due to many dangerous points in the oral cavity and laryngeal wall [60,63]. Over time, this decreases surgical therapy with curative intentions, which still remains as the best treatment. The diagnosis of low-grade dysplasia (SIN 1) is difficult and sometimes merges with a hyperplastic lesion. An amount of 20% of cases of hyperplastic lesions are true early dysplasia and need to be treated with curative intentions. At the end of the last century, the idea arose that these patients could be treated with medical therapy [3,8,64]. Costa A. et al. (1994) published that fenretinide N-(4-hydroxypheniyl) retinamide (4-HPR), an analog of vitamin A, was able to control and prevent the recurrences and new localization of oral dysplasia [65,66,67].

In 2005, F. Chiesa et al. conducted a clinical trial on the adjuvant chemotherapy of dysplastic lesions of the oral cavity using fenretinide. After one year of therapy, the authors found that fenretinide was effective in controlling recurrence but was associated with side effects [65]. Discriminating between NPDy and PDy and identifying cases that quickly progress to carcinoma versus those with delayed or no progression to carcinoma is now imperative. This work underscores the importance of transcriptomic analysis in dysplasia, suggesting that up-regulation of certain genes can help differentiate the latency of progression. This information is crucial for future medical therapy, especially in cases where surgical therapy fails to control the disease.

In our previous work, we demonstrated that TILs and gene alterations linked to TILs could assist physicians in selecting the most effective treatment, stratifying patients with high-risk evolving dysplasia based solely on TIL characteristics [5,10]. In this study, independent of TILs, we sought to assess transcriptional differences among patients with NDPy and PDy [5,10].

Among the 395 genes evaluated, certain genes were up-regulated or over-expressed, dividing the population in PDy and NPDy We focused on genes that showed statistical significance, derived from various classes such as chemokine signaling, B cell markers, lymphocyte infiltration, helper T cells, antigen processing, checkpoint inhibitors, neutrophils, and tumor markers (Table 2). The up-regulation of *CCL20* in NPDy has been associated with smoking habits [68]. Additionally, this gene imparts resistance to certain drugs such as 5-FU, platinum-based drugs, and some immunotherapeutic drugs used in HNSCC treatment [1,5,12,68,69]. In invasive carcinoma, *CCL20* has been reported to be up-regulated in association with fenretinide therapy (2.03-fold) and antifolate treatment, suggesting a synergistic action between *CCL20* and fenretinide and an inverse correlation with thymidylate synthetase (TS) [69,70,71]. In cases of methotrexate therapy (MTX), there is an up-regulation of *CCL20* together to TS reduction, increasing the pro-inflammatory response and secondary platinum sensitivity due to TS reduction [71].

We are aware that a high level of TS is responsible for drug treatment failure in laryngeal carcinoma, as reported in 2015 by Cossu Rocca et al. [72]. In cases of NPDy, an up-regulation of *CCL20* was observed, suggesting a potential therapeutic role for fenretinide, as reported in clinical trials by Chiesa F. et al. (2005), and the folate inhibitor drugs that improve treatment results [64,71,73]. These data help to identify dysregulated genes that could guide the best chance of therapeutic proposals as alternatives to surgery [64,73]. Of particular interest is that this gene, involving the immune system, alters neoplastic features and provides the ability to resist certain antineoplastic drugs.

While all literature works consistently demonstrated that *CCL20* up-regulation in invasive carcinoma, leading to a poor prognosis, the reasons behind its occurrence in dysplasia, particularly in association with NPDy, remain unknown. Notably, *RORC* is up-regulated in NPDy, whereas it has been reported to be down-regulated in carcinomas [71]. The interplay among ECM, tumors, and the inflammatory system plays a crucial role in the progression from dysplasia to carcinoma.

Studies by Lekva et al. (2013) have demonstrated that low levels of *RORC* in pituitary adenomas were associated with progression, tumor growth, and unfavorable response to treatment with somatostatin analog drugs [71]. Other studies showed a correlation between a low expression of *RORC* and a negative correlation with PD-L1 in bladder carcinoma, suggesting the suppression of the PD-L1/ITGB6 pathway [45,46,74]. The level of *RORC* has been identified as a predictor of prognosis and therapy response in bladder carcinoma. Additionally, a high intake of dietary retinoids has been linked to a 15% reduction in the incidence of bladder carcinoma [46,74]. Therefore, the association between *RORC* and fenretinide could be considered as a potential therapeutic strategy [45,46,74]. Given this, the possible role of fenretinide in the therapy of laryngeal dysplasia should be reappraised and highlighted through new transcriptional evaluations.

*CCR4* over-expression in PDy is correlated with platinum resistance and resistance to immunotherapeutic drugs such as mogamulizumab. The *CCR4* receptor is expressed by neoplastic cells and CCR4^+^Treg cells. CCR4^+^Treg cells modify the environment, allowing tumor cells to escape from the immune host response. Suppression of *CCR4* due to mogamulizumab, a monoclonal antibody IgG1-k, reduces the tumor burden and enhances antitumoral activity by decreasing CCR4^+^Treg cells and increasing the immune response [75,76]. In cases of PDy, we speculate that controlling the disease with mogamulizumab could be possible. Another option in these cases could be the potential association of mogamulizumab with another immunotherapeutic drug, such as nivolumab, as suggested by D.S. Hong et al. (2022) in their phase I/II study, although without an observed improvement in efficacy [77,78].

Of particular interest is the new T cell therapy for solid tumors, similar to the Car-T in hematological malignancies, which could be used in small dysplastic lesions of the larynx, especially when *CCL20* and *CCR4* are the most important gene alterations observed in dysplasia. [79,80]. In laboratory experiments, isolated cells expressing *CCL20* and *CCR4*, stimulated by IL-5 and IL-7, demonstrated an increased ability of CD8^+^ T cells to penetrate dysplastic cells, thereby improving therapeutic efficiency in fighting dysplasia. This therapy could be associated with mogamulizumab, an anti-CCR4 antibody, to amplify therapeutic efficiency. This immunotherapeutic drug has shown efficacy in T cell lymphomas [81].

PD-L2, encoded by the *PDCD1LG2* gene and belonging to checkpoint inhibitors that regulate the immune host response, has been found in APCs such as DCs, macrophages, helper, and cytotoxic T cells. It’s up-regulation in NPDy suggested a potential blockage of the host immune response, promoting neoplastic growth and tumor sensitivity to anti PD-L1 and PD-L2 drugs. We observed that this gene is up-regulated in cases of NPDy, suggesting an early phase of precocious blocking of the immune system response against neoplastic cell transformation. In this early phase, using immune drugs such as nivolumab could be a rational method to control dysplasia progression or delay or even recover from the disease with low side effects. A potential therapeutic approach involves the association of PD-L1 and PD-L2. Yearley et al. (2017) suggest that therapy is more effective when both PD-L1 and PD-L2 are positive. The prognosis and response to pembrolizumab were found to be greater when PD-L1 and PD-L2 were positive compared with PD-L1 positivity alone [48,78,82,83]. We observed that certain genes, which modify the host immune response, alter the tissue microenvironment (TME) acquiring staminal cell status by modifying the EMT. Our study indicated that the protective role of SIgs such as pIgA and pentameric IgM is lost when *JCHAIN* is up-regulated because there is a loss of SIg delivery through epithelial cells. Dysplastic cells are unable to transport pIgA and the pentameric IgM to the surface, compromising mucosal protection against infection. Infection in these cases could favor neoplastic progression due to mucosal inflammation, recruitment of inflammatory cells, and subsequent cellular damage [23,78,83,84]. A recent study by Junling Ren et al. (2023) showed that Porphyromonas Gengivalis infection promotes neoplastic growth by up-regulating the PD-L1 of DCs and CD8^+^ T cells, suggesting a potential medical therapy with anti-PD-L1. *JCHAIN*, reported to be up-regulated in patients responding to anti-PD-L1 therapy [85], raises speculation about the potential improvement of treatments with antibacterial topical or systemic therapy.

We underlined how various alterations linked to dysplasias can exist in the same patient. The standard treatment should be reappraised; while histopathological grading alone suggests possible treatments, mRNA transcriptional analysis provides interesting information about the stratus of the dysplasia and the potential cancer evolution. Distinguishing between NPDy and PDy in the future could lead to a change in therapy. The evaluation of targetable genes may reduce the need for re-treatment surgery and improve patients’ prognoses by selecting cases that require more aggressive therapy based on their transcriptional risk of malignant transformation.

We are aware of the potential for adverse events, the cost, and the lack of studies on the use of immune-focused drugs in the treatment of laryngeal dysplasia.

To date, there have been few studies on the use of drugs with a local focus, such as phototherapy [86]. In order to reduce costs and possible side effects, a local approach could only be chosen by planning specific prospective and multi-center clinical trials.

We acknowledge that this is a pilot study. While the evaluation involves only the mRNA transcripts and does not assess complete pathways and host–tumor interactions, it underscores a tight correlation with the immune system and tumor growth. This correlation may be due to poorly understood pathways involved in pre-neoplastic progression.

An additional point that differentiates our work from others is the selection of patients that all have laryngeal dysplasia. This is crucial because dysplastic lesions in the larynx differ from those in the oral cavity and do not exist for oropharyngeal carcinoma. We are aware that our study involved a small number of cases, implying low statistical power. Larger studies, including not only observational but also clinical trials that consider gene expression, should be planned for the future. Therefore, understanding how the immune system works and which genes are involved in the progression or delay of growth in LDy is pivotal for a better comprehension of the mechanisms controlling pre-neoplastic diseases in the head and neck.

## 4. Materials and Methods

We conducted a retrospective review of all patients treated with transoral laser microsurgery (TLM) between January 2005 and December 2020 in the Department of Otolaryngology and Head and Neck Surgery at the European Institute of Oncology (EIO), a tertiary comprehensive cancer center. All patients were referred to transoral laser microsurgery with radical intent, following the internal and current international NCCN standard of care [87].

Based on our previous doubled-matched case–control study, we considered 15 cases of LDy that evolved towards invasive LSCC during follow-up (PDy) and 31 cases of LDy that did not degenerate to LSCC (NPDy) for comparison [3].

The collected data included: age, sex, past medical history, pre-operative smoking and alcohol habits, site of the laryngeal lesion, surgical procedure adopted, histopathological findings, severity of squamous intraepithelial neoplasia (SIN), and progression to LSCC during follow-up.

Patients were excluded from the current study in cases of the following: previous LSCC, previous laryngeal surgery, immune diseases, or concurrent medical treatments interfering with immune system function (i.e., scleroderma, rheumatoid arthritis, steroid immunosuppressive treatments, etc.).

To comprehensively address the immune system factors and prognostic immunogenetic alterations for malignant progression, we assessed the presence of stromal tumor-infiltrating lymphocytes (TILs) in post-surgery specimens. Moreover, we used the RNA-based next-generation sequencing (NGS) panel Oncomine Immune Response Research Assay (OIRRA) (ThermoFisher, Waltham, MA, USA) to measure the expression of genes associated with lymphocyte regulation, cytokine signaling, lymphocyte markers, and checkpoint pathways in all cases of PDy. We also included two matched pair NPDy cases for each PDy case.

All patients signed an informed consent form for data use for scientific purposes, and the study was conducted in accordance with the Declaration of Helsinki and the guidelines provided by our ethical committee (ID Hospital trial: 2519).

### 4.1. Histopathological Analysis

We collected 46 cases of LDy from our surgical pathology department archive following the guidelines of our ethical committee. Standard staining with Hematoxylin and Eosin (H&E) was performed on formalin fixed paraffin-embedded blocks (FFPE) from the patients. The pathologists (FM, DL) evaluated the LDy grading following the system recommended by the International Agency Research on Cancer of the World Health Organization (IARC-WHO IARC 4th edition 2017) [88].

This system comprises three tailored grading levels: SIN 1 for low-grade dysplasia, SIN 2 for medium-grade dysplasia, and SIN 3 for high-grade dysplasia and carcinoma “in-situ”. This classification assesses the level of cell atypia within the epithelium. SIN 1 is characterized by the presence of dysplastic cells in the basal layer of the squamous epithelium, SIN 2 involves dysplastic alterations in the lower two-thirds of the epithelium while maintaining an upper level of maturation, and SIN 3 or “carcinoma in-situ” features dysplastic cells in all epithelial layers without any breaches in the basal lamina. According to this classification, SIN3 and “carcinoma in-situ” have the same high risk of malignant transformation, indicating a lesion that requires prompt treatment [88,89] (Table 2).

From each H&E stain, we studied TILs by selecting an area inclusive of dysplasia with underlying inflammatory cells (lymphocyte, macrophage, plasma cells avoiding the necrosis). Two pathologists (F.M. and D.L.) independently selected these areas in a double-blinded manner, following the methods outlined in our previous work [5].

For genomic analysis, we used the “NCBI-accession code” as the nucleotide analysis data base from GenBank^®^; we used the NIH genetic sequence database part of the International Nucleotide Sequence Database Collaboration, housed at the National Library Medicine (NLM), Bethesda, MD, USA [90].

### 4.2. Genetic Analysis

Of the 46 LDy specimens, we successfully extracted RNA transcript from 24 specimens, comprising 9 PDys and 15 NPDys. The RNA-based next-generation sequencing panel OIRRA (TermoFisher, Waltham, MA, USA) was used to assess the expression of 395 genes related to immune system activation. These genes included those associated with lymphocyte regulation, cytokine signaling, lymphocyte markers, checkpoint pathways, and tumor characterization. For the extraction, five unstained slides at 7 µm thicknesses were obtained from representative FFPE blocks. Manual microdissection was performed before nucleic acid isolation to selectively isolate dysplastic tissue. The RNA extraction was automatically performed with the Promega Maxwell instrument (Promega, Madison, WI, USA) using the Promega Maxwell RSC RNA FFPE kit. Sample purification was obtained using paramagnetic particles, providing a mobile solid phase for optimized sample capture, washing, and purification. Each sample was de-paraffinized in 300 μL of mineral oil for 2 min at 80 °C, followed by lysis with a master mix containing 224 μL of lysis buffer, 25 μL of proteinase K, and 1 μL of blue dye. After centrifugation at 10,000× *g* rpm for 30 s, samples were heated on a 56 °C heat block for 15 min and then transferred on an 80° heat block for one hour. Subsequently, each sample was treated with DNase cocktail containing 26 μL of MnCl2 (0.09 M), 14 μL of DNase Buffer, and 10 μL of reconstituted DNase I to catalyze the hydrolysis of DNA. Samples were incubated for 15 min at room temperature, then centrifuged at maximum speed for 5 min. Finally, the blue phase was transferred to well #1 of a Maxwell^®^ FFPE Cartridge. Each RNA sample was quantified using the QuantiFluor RNA system on the Quantus fluorometer. A quantitative RT-PCR was conducted in triplicate to determine the quality of the RNA samples using serial dilutions of the RNA standard (HL60 total RNA) for calibration (i.e., 50, 12.5, 3.13, 0.78, 0.2, and 0.05 ng/µL). Subsequently, each sample was treated with a reaction mix containing 8.75 μL of 4X TaqMan^®^ Fast Virus 1- Step Master Mix, 1.75 μL of 20X TaqMan^®^ Gene Expression Assay, GUSB, and 21 μL of nuclease-free water. An amount of 31.5 µL of the reaction mix and 3.5 µL of each sample, as well as standard and negative control (NTC), were added to a 96-well plate. An amount of 10 µL of each well was added to adjacent wells to obtain a triplicate. The plate was then sealed with a new MicroAmp™ Clear Adhesive Film (Life Technologies Quality Assurance, Carlsbad, CA, USA) and placed on 7900HT Fast Real-Time PCR System. Samples with a threshold value ≥0.2 were considered suitable for library preparation.

The RNA library preparation used an amplicon-based technology, enabling the sequencing of specific regions of interest (ROIs) only. Gene expression analysis was performed using the OIRRA NGS assay (Thermo Fisher Scientific, Waltham, MA, USA), targeting 395 immune-related genes. The RNA fragments underwent reverse transcription to cDNA using the SuperScript™ IV VILO™ (SSIV VILO) Master Mix (Thermo Fisher Scientific, Waltham, MA, USA). For each sample, 3 μL of 5X VILO™ Reaction Mix and 12 μL of total RNA (25 ng) 50 were dispensed onto Ion Code plates and loaded onto the thermal cycle. The final product was loaded onto the Ion Chef instrument (Thermo Fisher Scientific, Waltham, MA, USA) for automatized library amplification with the Ion AmpliSeq DL8 kit (ThermoFisher Scientific, Waltham, MA, USA) with specified thermal conditions (25 cycles for the amplification step, 4 min each). Libraries were then automatically loaded onto the Ion 530™ Chip and sequenced on Ion S5™ System (ThermoFisher Scientific, Waltham, MA, USA), following the manufacturer instructions. Primary analysis of the data was performed through the S5 torrent server. These data included chip well details, such as the percentage of ISP loading and enrichment, percentage of monoclonal and polyclonal DNA fragments, total number of reads, total bases, percentage of usable sequence meeting requirements for polyclonal fragments, low-quality and adapter dimers, as well as final library. Additionally, targeted RNA-sequencing data were analyzed using the Torrent Suite Immune Response RNA plugin, which produced gene transcript quantification from sequence read data.

For gene expression analysis, samples with mapped reads >1,000,000 and valid reads >800,000 were deemed adequate for further analysis. The data were processed using the Affymetrix Transcriptome Analysis Console software (TAC) v4.0. The gene expression sequencing data were transformed into logs and normalized for reads per million.

### 4.3. Statistical Analysis

Demographic and clinical characteristics were described using descriptive statistics based on PDy status, the main outcome measure. The only histopathological characteristic associated with the progression of dysplasia is the grading *p* = 0.029 (Table 2).

### 4.4. Gene Expression Analysis

RNA-sequencing data were obtained for the evaluation of gene expression levels. The gene expression read per million (RPM) data were centered log-ratio transformed after a non-parametric multivariate imputation of zeros for compositional data. After excluding one gene with low variability, data for 399 genes were available for this analysis. First, gene expression levels were categorized based on the presence or absence of expression. Second, gene expression levels were classified as “high” or “low” based on whether the expression level was higher or lower/equal to the sample median gene expression, respectively. Lastly, gene expression was considered on a continuous scale.

A heatmap was generated by performing a sparse partial least square differential analysis (sPLS-DA) with 7-fold cross-validation and 100 repeats. The most discriminative genes were selected based on the first and second component loading vectors using MixOmics Package 6.18.1. In particular, sPLS-DA was assessed using 7-fold cross-validation with 100 repeats.

Differences in gene expressions between PDy and NPDy were initially analyzed using univariate ANOVA tests, while univariate logistic regression models were also employed to assess the association of gene expression with PDy. Volcano plots were generated to visualize the results, presenting the beta coefficients obtained from ANOVA and logistic models against significance indicated by a *p*-value < 0.05.

In order to investigate the association with time to PDy, univariate Cox proportional hazards models were adopted, considering the sample median gene expression values as the cutoff. For genes found to be significantly associated with PDy, boxplots with Wilcoxon tests and survival curves with log-rank tests were presented (Supplementary Figures S1 and S2).

## Figures and Tables

**Table 1:** This table shows the genes involved in NPDy and PDy that results in a significant statistical value (*p* < 0.05), and for all genes, we can observe the function of genes associated with dysplasia; the nomenclature NCBI accession is: https://www.ncbi.nlm.nih.gov/nuccore/NM_005060.4 (accessed on 3 September 2024). (UR, up-regulated; OE, over-expression >2-fold).

NCBI-Accession CODE	Gene Function	PDy	NPDy	Gene
NM_004591	Chemokine signaling		UR	*CCL20*
NM_005508	Chemokine signaling	OE		*CCR4*
NM_000435	Tumor marker		UR	*NOTCH3*
NM_003068	Tumor marker steamness		UR	*SNAI2*
NM_002122	Antigen processing	OE		*HLA-DQA1*
NR_026972	Antigen-processing	OE		*HLA-F-AS1*
NM_001192	B cell marker	OE		*TNFRSF17*
NM_144646	B cell marker	OE		*JCHAIN*
NM_001783	B cell-receptor signaling	OE		*CD79A*
NM_025239	Checkpoint pathway		UR	*PDCD1LG2*
NM_003151	Helper T cell		UR	*STAT4*
NM_005060	Helper T cell		UR	*RORC*
NM_000717	Neutrophil	OE		*CA4*
NM_001780	Lymphocyte infiltrate	OE		*CD63*
NM_002727	Lymphocyte infiltrate	OE		*SRGN*
NM_004106	Lymphocyte infiltrate	OE		*FCER1G*

**Table 2:** Patient characteristics by PD status.

*p*-Value ^1^	PD	NPD	Characteristics
	n = 15 (100%)	n = 31 (100%)	
0.2			Sex
	1 (6.7%)	8 (26%)	Female
	14 (93%)	23 (74%)	Male
0.2	66 (56, 70)	58 (54, 64)	Age, median (IQR)
0.8			Smoking
	3 (20%)	4 (13%)	Never
	6 (40%)	13 (42%)	Current
	6 (40%)	14 (45%)	Former
0.6			Cancer site
	13 (87%)	27 (87%)	Glottis
	0 (0%)	2 (6.5%)	Supraglottis
	2 (13%)	2 (6.5%)	Glottis + Supraglottis
0.2			Side
	11 (73%)	28 (90%)	Monolateral
	4 (27%)	3 (9.7%)	Bilateral
0.029			Histological grade
	3 (20%)	11 (35%)	SIN 1
	1 (6.7%)	10 (32%)	SIN 2
	11 (73%)	10 (32%)	SIN 3
0.4			Multifocal
	11 (73%)	26 (84%)	No
	4 (27%)	5 (16%)	Yes

^1^ *p*-values: Fisher’s exact or chi-square test or Wilcoxon rank sum test for the continuous variable: we are grateful to Cancers for their kindly concession to use this picture.

## Data Availability

The data presented in this study are available upon request from the corresponding author.

## Supplementary Materials

The following supporting information can be downloaded at: https://www.mdpi.com/article/10.3390/ijms25179685/s1.

## References

[1] Califano J., Van Der Riet P., Westra W., Nawroz H., Clayman G., Piantadosi S., Corio R., Lee D., Greenberg B., Koch W. (1996). Genetic progression model for head and neck cancer: Implications for field cancerization. Cancer Res..

[2] Califano J., Westra W.H., Meininger G., Corio R., Koch W.M., Sidransky D. (2000). Genetic progression and clonal relationship of recurrent premalignant head and neck lesions. Clin. Cancer Res. Off. J. Am. Assoc. Cancer Res..

[3] Chu F., De Santi S., Tagliabue M., De Benedetto L., Zorzi S., Pietrobon G., Herman I., Maffini F., Chiocca S., Corso F. (2021). Laryngeal dysplasia: Oncological outcomes in a large cohort of patients treated in a tertiary comprehensive cancer centre. Am. J. Otolaryngol..

[4] Bae J.Y., Choi K.U., Kim A., Lee S.J., Kim K., Kim J.Y., Lee I.S., Chung S.H., Kim J.I. (2020). Evaluation of immune-biomarker expression in high-grade soft-tissue sarcoma: HLA-DQA1 expression as a prognostic marker. Exp. Ther. Med..

[5] Chu F., Maffini F., Lepanto D., Vacirca D., Taormina S.V., De Berardinis R., Gandini S., Vignati S., Ranghiero A., Rappa A. (2023). The Genetic and Immunologic Landscape Underlying the Risk of Malignant Progression in Laryngeal Dysplasia. Cancers.

[6] Arsenic R., Kurrer M.O. (2013). Differentiated dysplasia is a frequent precursor or associated lesion in invasive squamous cell carcinoma of the oral cavity and pharynx. Virchows Arch..

[7] Van Hulst A.M., Kroon W., van der Linden E.S., Nagtzaam L., Ottenhof S.R., Wegner I., Gunning A.C., Grolman W., Braunius W. (2016). Grade of dysplasia and malignant transformation in adults with premalignant laryngeal lesions. Head. Neck..

[8] Lee D.H., Yoon T.M., Lee J.K., Lim S.C. (2015). Predictive factors of recurrence and malignant transformation in vocal cord leukoplakia. Eur. Arch. Otorhinolaryngol..

[9] Thompson L.D.R. (2017). Laryngeal Dysplasia, Squamous Cell Carcinoma, and Variants. Surg. Pathol. Clin..

[10] Tagliabue M., Maffini F., Fumagalli C., Gandini S., Lepanto D., Corso F., Cacciola S., Ranghiero A., Rappa A., Vacirca D. (2020). A role for the immune system in advanced laryngeal cancer. Sci. Rep..

[11] Fan T., Li S., Xiao C., Tian H., Zheng Y., Liu Y., Li C., He J. (2022). CCL20 promotes lung adenocarcinoma progression by driving epithelial-mesenchymal transition. Int. J. Biol. Sci..

[12] Song Q., Shang J., Zhang C., Chen J., Zhang L., Wu X. (2020). Transcription factor RUNX3 promotes CD8+ T cell recruitment by CCL3 and CCL20 in lung adenocarcinoma immune microenvironment. J. Cell Biochem..

[13] Kadomoto S., Izumi K., Mizokami A. (2020). The CCL20-CCR6 Axis in Cancer Progression. Int. J. Mol. Sci..

[14] Lörchner H., Cañes Esteve L., Góes M.E., Harzenetter R., Brachmann N., Gajawada P., Günther S., Doll N., Pöling J., Braun T. (2023). Neutrophils for Revascularization Require Activation of CCR6 and CCL20 by TNFα. Circ. Res..

[15] Ling Z., Li W., Hu J., Li Y., Deng M., Zhang S., Ren X., Wu T., Xia J., Cheng B. (2022). Targeting CCL2-CCR4 axis suppress cell migration of head and neck squamous cell carcinoma. Cell Death Dis..

[16] Tsai S.C., Sheen M.C., Chen B.H. (2011). Association between HLA-DQA1, HLA-DQB1 and oral cancer. Kaohsiung J. Med. Sci..

[17] Huang Y., Sun H., Ma X., Zeng Y., Pan Y., Yu D., Liu Z., Xiang Y. (2020). HLA-F-AS1/miR-330-3p/PFN1 axis promotes colorectal cancer progression. Life Sci..

[18] Gao S.J., Ren S.N., Liu Y.T., Yan H.W., Chen X.B. (2021). Targeting EGFR sensitizes 5-Fu-resistant colon cancer cells through modification of the lncRNA-FGD5-AS1-miR-330-3p-Hexokinase 2 axis. Mol. Ther. Oncolytics..

[19] Lim K.H., Yang Y., Staudt L.M. (2012). Pathogenetic importance and therapeutic implications of NF-κB in lymphoid malignancies. Immunol. Rev..

[20] Johansen F.E., Braathen R., Brandtzaeg P. (2000). Role of J chain in secretory immunoglobulin formation. Scand. J. Immunol..

[21] Chen Y., Jiang N., Chen M., Sui B., Liu X. (2022). Identification of tumor antigens and immune subtypes in head and neck squamous cell carcinoma for mRNA vaccine development. Front. Cell Dev. Biol..

[22] Ting H.S.L., Chen Z., Chan J.Y.K. (2023). Systematic review on oral microbial dysbiosis and its clinical associations with head and neck squamous cell carcinoma. Head Neck.

[23] Pan J., Weng Z., Xue C., Lin B., Lin M. (2021). The Bioinformatics-Based Analysis Identifies 7 Immune-Related Genes as Prognostic Biomarkers for Colon Cancer. Front. Oncol..

[24] Mancardi D.A., Albanesi M., Jönsson F., Iannascoli B., Van Rooijen N., Kang X., England P., Daëron M., Bruhns P. (2013). The high-affinity human IgG receptor FcγRI (CD64) promotes IgG-mediated inflammation, anaphylaxis, and antitumor immunotherapy. Blood.

[25] Mladenov R., Hristodorov D., Cremer C., Gresch G., Grieger E., Schenke L., Klose D., Amoury M., Woitok M., Jost E. (2016). CD64-directed microtubule associated protein tau kills leukemic blasts ex vivo. Oncotarget.

[26] Chou C., Zhang X., Krishna C., Nixon B.G., Dadi S., Capistrano K.J., Kansler E.R., Steele M., Han J., Shyu A. (2022). Programme of self-reactive innate-like T cell-mediated cancer immunity. Nature.

[27] Dadi S., Chhangawala S., Whitlock B.M., Franklin R.A., Luo C.T., Oh S.A., Toure A., Pritykin Y., Huse M., Leslie C.S. (2016). Cancer Immunosurveillance by Tissue-Resident Innate Lymphoid Cells and Innate-like T Cells. Cell.

[28] Fu L., Cheng Z., Dong F., Quan L., Cui L., Liu Y., Zeng T., Huang W., Chen J., Pang Y. (2020). Enhanced expression of FCER1G predicts positive prognosis in multiple myeloma. J. Cancer.

[29] Andreu P., Johansson M., Affara N.I., Pucci F., Tan T., Junankar S., Korets L., Lam J., Tawfik D., DeNardo D.G. (2010). FcRgamma activation regulates inflammation-associated squamous carcinogenesis. Cancer Cell.

[30] Zhang X., Cai J., Song F., Yang Z. (2022). Prognostic and immunological role of FCER1G in pan-cancer. Pathol. Res. Pract..

[31] Mantovani A., Allavena P., Sica A., Balkwill F. (2008). Cancer-related inflammation. Nature.

[32] Radford K.J., Thorne R.F., Hersey P. (1997). Regulation of tumor cell motility and migration by CD63 in a human melanoma cell line. J. Immunol..

[33] Chen Z., Mustafa T., Trojanowicz B., Brauckhoff M., Gimm O., Schmutzler C., Köhrle J., Holzhausen H., Kehlen A., Klonisch T. (2004). CD82, and CD63 in thyroid cancer. Int. J. Mol. Med..

[34] Jang H.I., Lee H. (2003). A decrease in the expression of CD63 tetraspanin protein elevates invasive potential of human melanoma cells. Exp. Mol. Med..

[35] Takino T., Miyamori H., Kawaguchi N., Uekita T., Seiki M., Sato H. (2003). Tetraspanin CD63 promotes targeting and lysosomal proteolysis of membrane-type 1 matrix metalloproteinase. Biochem. Biophys. Res. Commun..

[36] Wang Y.L., Ren D., Lu J.L., Jiang H., Wei J.Z., Lan J., Liu F., Qu S.H. (2022). STAT3 regulates SRGN and promotes metastasis of nasopharyngeal carcinoma through the FoxO1-miR-148a-5p-CREB1 axis. Lab. Investig..

[37] Nava V.E., Khosla R., Shin S., Mordini F.E., Bandyopadhyay B.C. (2022). Enhanced carbonic anhydrase expression with calcification and fibrosis in bronchial cartilage during COPD. Acta Histochem..

[38] Liu Z., Bai Y., Xie F., Miao F., Du F. (2020). Comprehensive Analysis for Identifying Diagnostic and Prognostic Biomarkers in Colon Adenocarcinoma. DNA Cell Biol..

[39] Thierfelder W.E., van Deursen J.M., Yamamoto K., Tripp R.A., Sarawar S.R., Carson R.T., Sangster M.Y., Vignali D.A., Doherty P.C., Grosveld G.C. (1996). Requirement for Stat4 in interleukin-12-mediated responses of natural killer and T cells. Nature.

[40] Bacon C.M., Petricoin E.F., Ortaldo J.R., Rees R.C., Larner A.C., Johnston J.A., O’Shea J.J. (1995). Interleukin 12 Induces Tyrosine Phosphorylation and Activation of STAT4 in Human Lymphocytes. Proc. Natl. Acad. Sci. USA.

[41] Yamamoto K., Quelle F.W., Thierfelder W.E., Kreider B.L., Gilbert D.J., Jenkins N.A., Copeland N.G., Silvennoinen O., Ihle J.N. (1994). Stat4, a novel gamma interferon activation site-binding protein expressed in early myeloid differentiation. Mol. Cell Biol..

[42] Wiche Salinas T.R., Zhang Y., Sarnello D., Zhyvoloup A., Marchand L.R., Fert A., Planas D., Lodha M., Chatterjee D., Karwacz K. (2021). Th17 cell master transcription factor RORC2 regulates HIV-1 gene expression and viral outgrowth. Proc. Natl. Acad. Sci. USA.

[43] Villey I., de Chasseval R., de Villartay J.-P. (1999). RORγT, a thymus-specific isoform of the orphan nuclear receptor RORγ / TOR, is up-regulated by signaling through the pre-T cell receptor and binds to the TEA promoter. Eur. J. Immunol..

[44] Marchenko S., Piwonski I., Hoffmann I., Sinn B.V., Kunze C.A., Monjé N., Pohl J., Kulbe H., Schmitt W.D., Darb-Esfahani S. (2023). Prognostic value of regulatory T cells and T helper 17 cells in high grade serous ovarian carcinoma. J. Cancer Res. Clin. Oncol..

[45] Tratnjek L., Jeruc J., Romih R., Zupančič D. (2021). Vitamin A and Retinoids in Bladder Cancer Chemoprevention and Treatment: A Narrative Review of Current Evidence, Challenges and Future Prospects. Int. J. Mol. Sci..

[46] Cao D., Qi Z., Pang Y., Li H., Xie H., Wu J., Huang Y., Zhu Y., Shen Y., Zhu Y. (2019). Retinoic Acid-Related Orphan Receptor C Regulates Proliferation, Glycolysis, and Chemoresistance via the PD-L1/ITGB6/STAT3 Signaling Axis in Bladder Cancer. Cancer Res..

[47] Battula V.L., Evans K.W., Hollier B.G., Shi Y., Marini F.C., Ayyanan A., Wang R.Y., Brisken C., Guerra R., Andreeff M. (2010). Epithelial-mesenchymal transition-derived cells exhibit multilineage differentiation potential similar to mesenchymal stem cells. Stem Cells.

[48] Yearley J.H., Gibson C., Yu N., Moon C., Murphy E., Juco J., Lunceford J., Cheng J., Chow L.Q.M., Seiwert T.Y. (2017). PD-L2 Expression in Human Tumors: Relevance to Anti-PD-1 Therapy in Cancer. Clin. Cancer Res..

[49] Moratin J., Metzger K., Safaltin A., Herpel E., Hoffmann J., Freier K., Hess J., Horn D. (2019). Upregulation of PD-L1 and PD-L2 in neck node metastases of head and neck squamous cell carcinoma. Head Neck.

[50] Bossi P., Siano M., Bergamini C., Cossu Rocca M., Sponghini A.P., Giannoccaro M., Tonella L., Paoli A., Marchesi E., Perrone F. (2017). Are Fusion Transcripts in Relapsed/Metastatic Head and Neck Cancer Patients Predictive of Response to Anti-EGFR Therapies?. Dis. Markers.

[51] Hosseini-Alghaderi S., Baron M. (2020). Notch3 in Development, Health and Disease. Biomolecules.

[52] Zhang Y.Q., Liang Y.K., Wu Y., Chen M., Chen W.L., Li R.H., Zeng Y.Z., Huang W.H., Wu J.D., Zeng D. (2021). Notch3 inhibits cell proliferation and tumorigenesis and predicts better prognosis in breast cancer through transactivating PTEN. Cell Death Dis..

[53] Aburjania Z., Jang S., Whitt J., Jaskula-Stzul R., Chen H., Rose J.B. (2018). The Role of Notch3 in Cancer. Oncologist.

[54] Man C.H., Wei-Man Lun S., Wai-Ying Hui J., To K.F., Choy K.W., Wing-Hung Chan A., Chow C., Tin-Yun Chung G., Tsao S.W., Tak-Chun Yip T. (2012). Inhibition of NOTCH3 signalling significantly enhances sensitivity to cisplatin in EBV-associated nasopharyngeal carcinoma. J. Pathol..

[55] Zhang T.H., Liu H.C., Zhu L.J., Chu M., Liang Y.J., Liang L.Z., Liao G.Q. (2011). Activation of Notch signaling in human tongue carcinoma. J. Oral. Pathol. Med..

[56] Wang H., Chirshev E., Hojo N., Suzuki T., Bertucci A., Pierce M., Perry C., Wang R., Zink J., Glackin C.A. (2021). The Epithelial-Mesenchymal Transcription Factor SNAI1 Represses Transcription of the Tumor Suppressor miRNA let-7 in Cancer. Cancers.

[57] Liu X., Zhang N., Chen Q., Feng Q., Zhang Y., Wang Z., Yue X., Li H., Cui N. (2023). SNAI2 Attenuated the Stem-like Phenotype by Reducing the Expansion of EPCAMhigh Cells in Cervical Cancer Cells. Int. J. Mol. Sci..

[58] Gan C.P., Lee B.K.B., Lau S.H., Kallarakkal T.G., Zaini Z.M., Lye B.K.W., Zain R.B., Sathasivam H.P., Yeong J.P.S., Savelyeva N. (2022). Transcriptional analysis highlights three distinct immune profiles of high-risk oral epithelial dysplasia. Front. Immunol..

[59] Slaughter D.P., Southwick H.W., Smejkal W. (1953). Field cancerization in oral stratified squamous epithelium; clinical implications of multicentric origin. Cancer.

[60] Tang Y., Weiss S.J. (2017). Snail/Slug-YAP/TAZ complexes cooperatively regulate mesenchymal stem cell function and bone formation. Cell Cycle.

[61] Tabor M.P., Braakhuis B.J., van der Wal J.E., van Diest P.J., Leemans C.R., Brakenhoff R.H., Kummer J.A. (2003). Comparative molecular and histological grading of epithelial dysplasia of the oral cavity and the oropharynx. J. Pathol..

[62] González M.V., Pello M.F., López-Larrea C., Suárez C., Menéndez M.J., Coto E. (1995). Loss of heterozygosity and mutation analysis of the p16 (9p21) and p53 (17p13) genes in squamous cell carcinoma of the head and neck. Clin. Cancer Res..

[63] Jang S.J., Chiba I., Hirai A., Hong W.K., Mao L. (2001). Multiple oral squamous epithelial lesions: Are they genetically related?. Oncogene.

[64] O’Shaughnessy J.A., Kelloff G.J., Gordon G.B., Dannenberg A.J., Hong W.K., Fabian C.J., Sigman C.C., Bertagnolli M.M., Stratton S.P., Lam S. (2002). Treatment and prevention of intraepithelial neoplasia: An important target for accelerated new agent development. Clin. Cancer Res..

[65] Chiesa F., Tradati N., Grigolato R., Boracchi P., Biganzoli E., Crose N., Cavadini E., Formelli F., Costa L., Giardini R. (2005). Randomized trial of fenretinide (4-HPR) to prevent recurrences, new localizations and carcinomas in patients operated on for oral leukoplakia: Long-term results. Int. J. Cancer.

[66] Costa A., Formelli F., Chiesa F., Decensi A., De Palo G., Veronesi U. (1994). Prospects of chemoprevention of human cancers with the synthetic retinoid fenretinide. Cancer Res..

[67] Tradati N., Chiesa F., Rossi N., Grigolato R., Formelli F., Costa A., de Palo G. (1994). Successful topical treatment of oral lichen planus and leukoplakias with fenretinide (4-HPR). Cancer Lett..

[68] Wang G.Z., Cheng X., Li X.C., Liu Y.Q., Wang X.Q., Shi X., Wang Z.Y., Guo Y.Q., Wen Z.S., Huang Y.C. (2015). Tobacco smoke induces production of chemokine CCL20 to promote lung cancer. Cancer Lett..

[69] Ferrari N., Pfeffer U., Dell’Eva R., Ambrosini C., Noonan D.M., Albini A. (2005). The transforming growth factor-beta family members bone morphogenetic protein-2 and macrophage inhibitory cytokine-1 as mediators of the antiangiogenic activity of N-(4-hydroxyphenyl)-retinamide. Clin. Cancer Res..

[70] Mlynska A., Salciuniene G., Zilionyte K., Garberyte S., Strioga M., Intaite B., Barakauskiene A., Lazzari G., Dobrovolskiene N., Krasko J.A. (2019). Chemokine profiling in serum from patients with ovarian cancer reveals candidate biomarkers for recurrence and immune infiltration. Oncol. Rep..

[71] Lekva T., Berg J.P., Heck A., Lyngvi Fougner S., Olstad O.K., Ringstad G., Bollerslev J., Ueland T. (2013). Attenuated RORC expression in the presence of EMT progression in somatotroph adenomas following treatment with somatostatin analogs is associated with poor clinical recovery. PLoS ONE.

[72] Cossu Rocca M., Maffini F., Chiocca S., Massaro M., Santoro L., Cattaneo A., Verri E., Chiesa F., Preda L., Nole F. (2015). Induction chemotherapy followed by transoral laser microsurgery: A mutimodal approach to improve outcomes for locally advanced laryngeal cancer patients?. J. Clin. Oncol..

[73] Municio C., Soler Palacios B., Estrada-Capetillo L., Benguria A., Dopazo A., García-Lorenzo E., Fernández-Arroyo S., Joven J., Miranda-Carús M.E., González-Álvaro I. (2016). Methotrexate selectively targets human proinflammatory macrophages through a thymidylate synthase/p53 axis. Ann. Rheum. Dis..

[74] Wu S., Liu Y., Michalek J.E., Mesa R.A., Parma D.L., Rodriguez R., Mansour A.M., Svatek R., Tucker T.C., Ramirez A.G. (2020). Carotenoid Intake and Circulating Carotenoids Are Inversely Associated with the Risk of Bladder Cancer: A Dose-Response Meta-analysis. Adv Nutr..

[75] Ishida T., Ueda R. (2006). CCR4 as a novel molecular target for immunotherapy of cancer. Cancer Sci..

[76] Zhang T., Sun J., Li J., Zhao Y., Zhang T., Yang R., Ma X. (2021). Safety and efficacy profile of mogamulizumab (Poteligeo) in the treatment of cancers: An update evidence from 14 studies. BMC Cancer.

[77] Mollica Poeta V., Massara M., Capucetti A., Bonecchi R. (2019). Chemokines and Chemokine Receptors: New Targets for Cancer Immunotherapy. Front. Immunol..

[78] Hong D.S., Rixe O., Chiu V.K., Forde P.M., Dragovich T., Lou Y., Nayak-Kapoor A., Leidner R., Atkins J.N., Collaku A. (2022). Mogamulizumab in Combination with Nivolumab in a Phase I/II Study of Patients with Locally Advanced or Metastatic Solid Tumors. Clin. Cancer Res..

[79] Zhong C., Chen J. (2021). CAR-T cell engineering with CCR6 exhibits superior anti-solid tumor efficacy. Sci. Bull..

[80] Jin L., Cao L., Zhu Y., Cao J., Li X., Zhou J., Liu B., Zhao T. (2021). Enhance anti-lung tumor efficacy of chimeric antigen receptor-T cells by ectopic expression of C-C motif chemokine receptor 6. Sci. Bull..

[81] Ueda R. (2015). Clinical Application of Anti-CCR4 Monoclonal Antibody. Oncology.

[82] Zhang Y., Chung Y., Bishop C., Daugherty B., Chute H., Holst P., Kurahara C., Lott F., Sun N., Welcher A.A. (2006). Regulation of T cell activation and tolerance by PDL2. Proc. Natl. Acad. Sci. USA.

[83] Pardoll D.M. (2012). The blockade of immune checkpoints in cancer immunotherapy. Nat. Rev. Cancer.

[84] Zhang L., Liu Y., Zheng H.J., Zhang C.P. (2020). The Oral Microbiota May Have Influence on Oral Cancer. Front. Cell Infect. Microbiol..

[85] Ren J., Han X., Lohner H., Hoyle R.G., Li J., Liang S., Wang H.P. (2023). gingivalis Infection Upregulates PD-L1 Expression on Dendritic Cells, Suppresses CD8+ T-cell Responses, and Aggravates Oral Cancer. Cancer Immunol. Res..

[86] Kübler A., Haase T., Rheinwald M., Barth T., Mühling J. (1998). Treatment of oral leukoplakia by topical application of 5-aminolevulinic acid. Int. J. Oral Maxillofac. Surg..

[87] National Comprehensive Cancer Network. https://www.nccn.org/guidelines/recently-published-guidelines.

[88] Gale N., Hille J., Jordan R.C., Nadal A., William M.D. (2017). WHO Classification of Head and Neck Tumor.

[89] Zhang H.K., Liu H.G. (2012). Is severe dysplasia the same lesion as carcinoma in situ? 10-Year follow-up of laryngeal precancerous lesions. Acta Otolaryngol..

[90] GenBank ^®^—The NIH Genetic Sequence Database Part of the International Nucleotide Sequence Database Collaboration. Housed at the National Library Medicine (NLM), Bethesda, MD, USA. https://www.ncbi.nlm.nih.gov/nuccore.

