# Supplementary material for: A Transcriptomic Analysis of Laryngeal Dysplasia
Source: Int J Mol Sci. 2024 Sep 7;25(17):9685. doi: 10.3390/ijms25179685 (PMC11395940; doi:10.3390/ijms25179685)
Supplement: Supplementary file 1 [file ijms-25-09685-s001.zip › ijms-3159924-supplementary.pdf]

Supplementary Figures:

Supplementary Figure S1. The box-plot illustrating the distribution of genes able to discriminate between PDy and NPDy.

S1

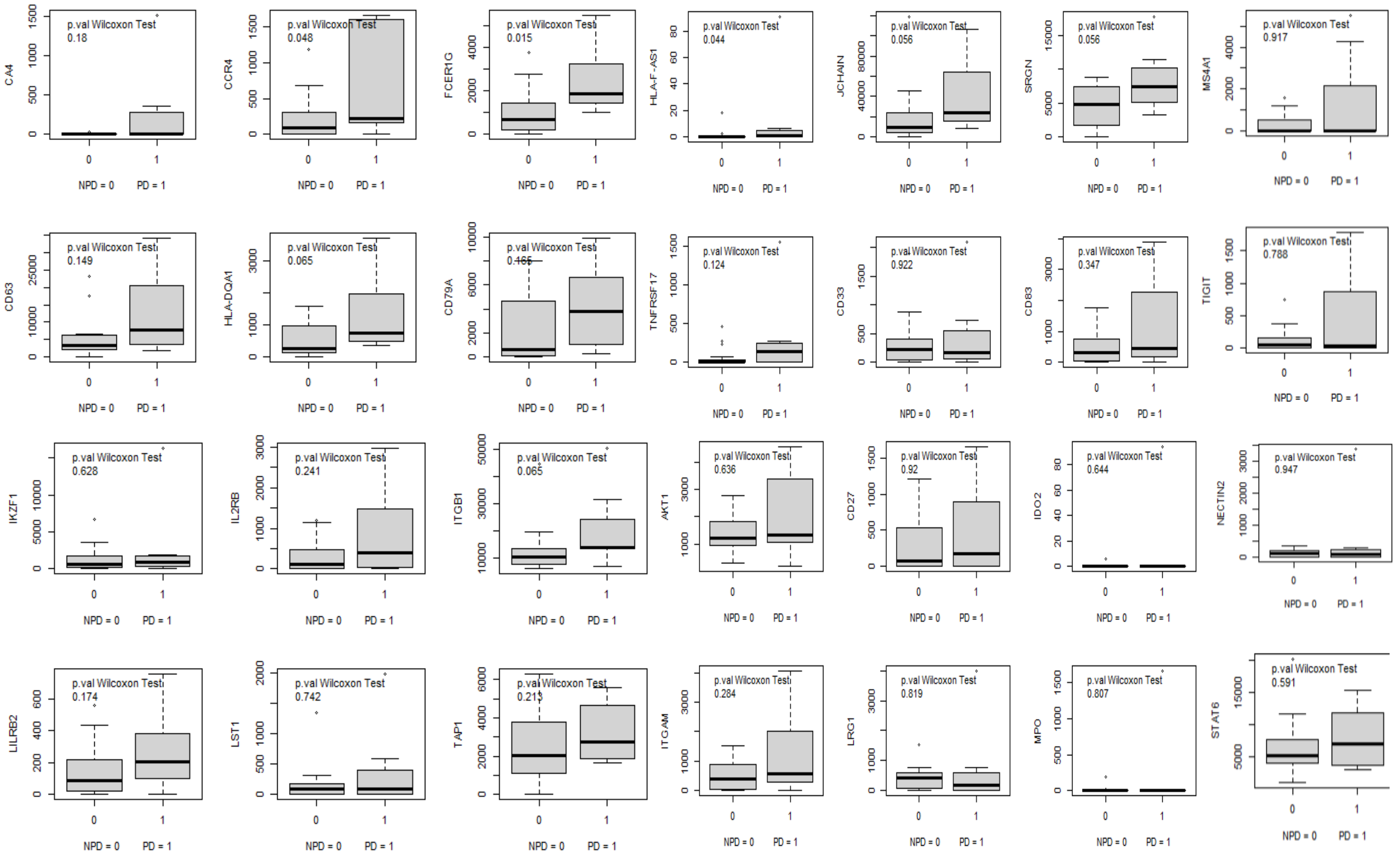

Supplementary Figure S2. A box plot illustrating the distribution of genes derived from the ANOVA analysis up-regulated in the PDy group

S2

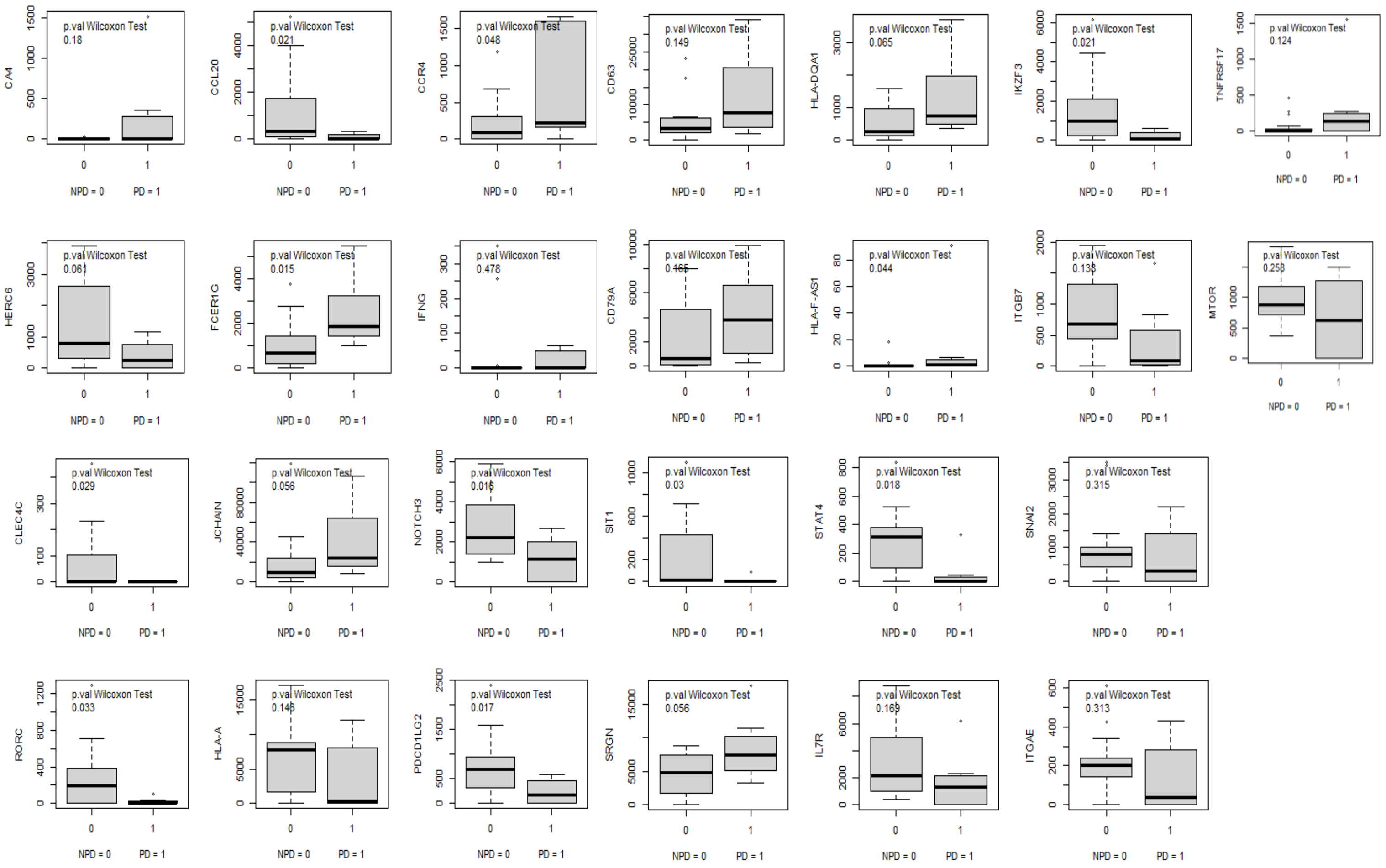

Supplementary S3:

Method used to perfom a Keratin 13 staining.

Clone: Anti-Cytokeratin 13 antibody [AE8]

Description: Mouse monoclonal [AE8] to Cytokeratin 13

Factory: ABCAM (trade mark)

Cytokeratin 13 staining was performed after formalin-fixed paraffin-embedded tissue: The section of 5 microns thigh was pre-treated using heat mediated antigen retrieval with sodium citrate buffer (pH 6, epitope retrieval solution 1) for 20 mins. The section was then incubated with keratin 13 antibody, 0.05 µg/ml, for 15 mins at room temperature and detected using an HRP conjugated compact polymer system. DAB was used as the chromogen. The section was then counterstained with hematoxylin and mounted with DPX.

For other IHC staining systems (automated and non-automated) customers should optimize variable parameters such as antigen retrieval conditions, primary antibody concentration and antibody incubation times following the instruction Datasheet
